# Generalising XTRACT tractography protocols across common macaque brain templates

**DOI:** 10.1007/s00429-024-02760-0

**Published:** 2024-02-23

**Authors:** Stephania Assimopoulos, Shaun Warrington, Katherine L. Bryant, Stefan Pszczolkowski, Saad Jbabdi, Rogier B. Mars, Stamatios N. Sotiropoulos

**Affiliations:** 1 Sir Peter Mansfield Imaging Centre, Mental Health and Clinical Neurosciences, School of Medicine, University of Nottingham, Nottingham, UK; 2 Laboratoire de Psychologie Cognitive, Aix-Marseille Université, Marseille, France; 3 Wellcome Centre for Integrative Neuroimaging (WIN-FMRIB), Nuffield Department of Clinical Neurosciences, University of Oxford, Oxford, UK; 4 NIHR Nottingham Biomedical Research Centre, University of Nottingham, Nottingham, UK; 5 Donders Institute for Brain, Cognition and Behaviour, Radboud University, Nijmegen, The Netherlands

**Keywords:** Diffusion MRI, Connectivity, NHP, F99, NMT, Yerkes19, INIA, Comparative anatomy

## Abstract

Non-human primates are extensively used in neuroscience research as models of the human brain, with the rhesus macaque being a prominent example. We have previously introduced a set of tractography protocols (XTRACT) for reconstructing 42 corresponding white matter (WM) bundles in the human and the macaque brain and have shown cross-species comparisons using such bundles as WM landmarks. Our original XTRACT protocols were developed using the F99 macaque brain template. However, additional macaque template brains are becoming increasingly common. Here, we generalise the XTRACT tractography protocol definitions across five macaque brain templates, including the F99, D99, INIA, Yerkes and NMT. We demonstrate equivalence of such protocols in two ways: (a) Firstly by comparing the bodies of the tracts derived using protocols defined across the different templates considered, (b) Secondly by comparing the projection patterns of the reconstructed tracts across the different templates in two cross-species (human–macaque) comparison tasks. The results confirm similarity of all predictions regardless of the macaque brain template used, providing direct evidence for the generalisability of these tractography protocols across the five considered templates.

## Introduction

Non-human primates (NHPs) are extensively used in neuroscience research to explore the effects of evolution of the brain and its organisation, and as models of the human brain, with the rhesus macaque being a prominent example. This is possible because several neuroanatomical features and organisational principles are evolutionarily conserved across higher primates, including the presence of major white matter (WM) tracts (Markov et al. 2014; Petrides and Pandya 1999; Thiebaut de Schotten et al. 2012). For instance, several association pathways have been identified in humans, chimpanzees, and macaques, connecting similar broad brain areas, whilst differing in their branching patterns, as a result of ecological adaptation (connectivity specialisation) (Bryant et al. 2020; Eichert et al. 2020; Hecht et al. 2013; Petrides and Pandya 2009; Rilling et al. 2008). WM organisation is of particular functional interest as each functional brain subunit can be uniquely identified by its pattern of its extrinsic WM connections (Passingham et al. 2002).Therefore, mapping connectivity patterns in the NHP brain is of great interest, as it can provide insight into the human brain (Mars et al. 2018b).

Tracing (chemical, viral etc.) provides a gold standard (Jbabdi et al. 2015; Lehman et al. 2011) towards mapping brain connectivity, however, it is limited due to its invasiveness, and in its scalability, whole-brain applicability and translatability in humans. Magnetic resonance imaging (MRI) provides an alternative to invasive brain mapping, in both the human and NHP brain. Diffusion MRI (dMRI) specifically is a unique tool for identifying structural connections in the brain (Jbabdi et al. 2015). Various dMRI-based tractography methods have been developed to reliably extract defined sets of WM tracts. Such approaches typically use tractography protocols which consist of constraints/rules to guide the curve propagation process, reflecting underlying anatomy (Catani et al. 2002; Wakana et al. 2004). Protocols must be robust, consistent and reproducible whilst still reflecting the natural anatomical variation amongst individuals. This can be achieved in two ways, either manually prescribing subject-specific protocols (labour intensive especially for large cohorts; reduced consistency in protocol definition) or defining them in a standardised template space and mapping to the individual subjects in a consistent and automated fashion. The latter approach has proven to be rather powerful in the extraction of a considerable range of tracts, with a particular advantage when applied to large cohorts as it has allowed for the development of tract-specific atlases and the extraction of tract-specific features (Catani et al. 2012; de Schotten et al. 2011; Eichert et al. 2019; Makris et al. 2009; Takemura et al. 2017; Zhao et al. 2016).

**Fig. 1:**
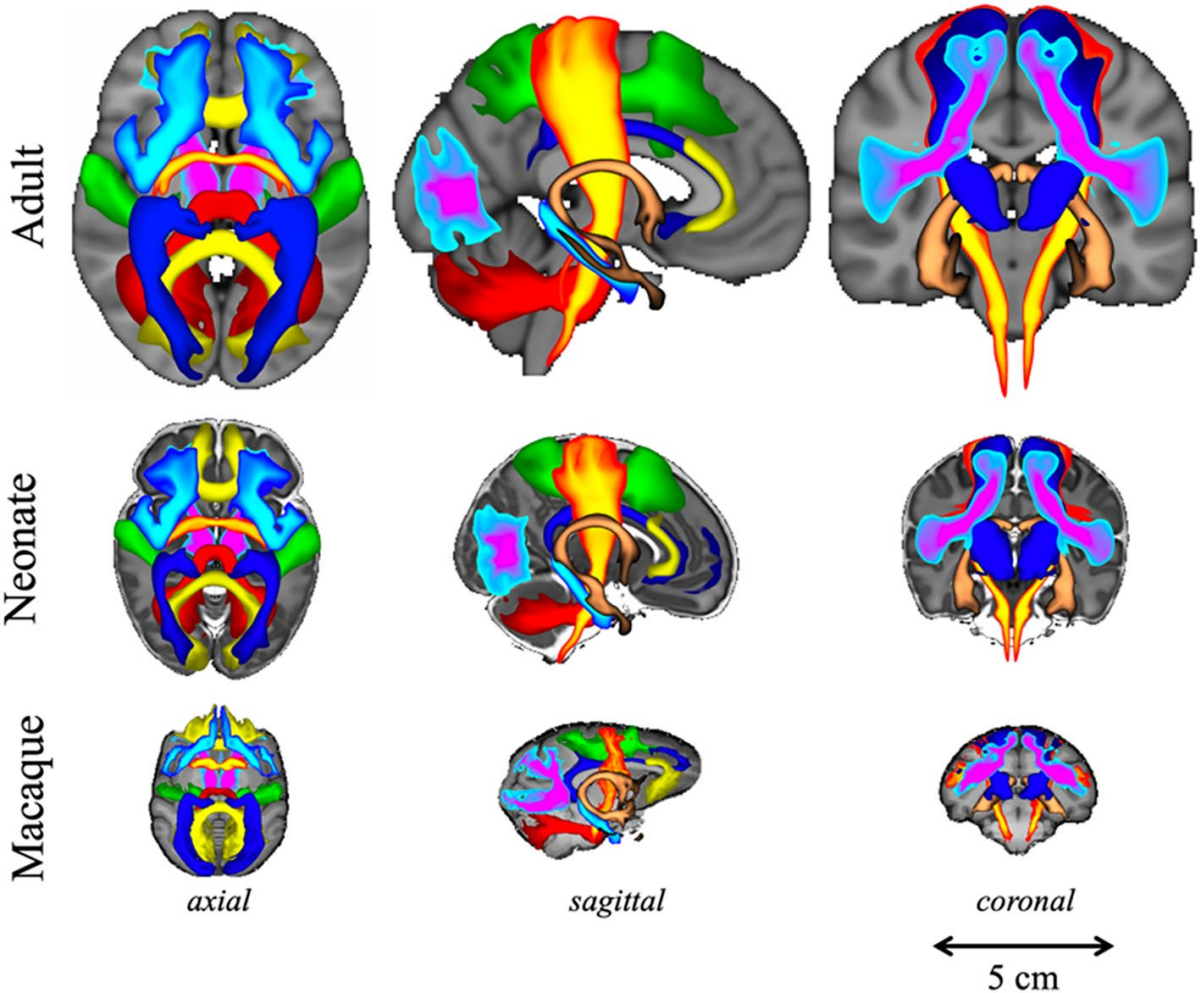
Axial, sagittal, and coronal views of population percentage atlases of a subset of the 42 XTRACT WM bundles for the human (adult and neonate) and the macaque brain. Reproduced from (Warrington et al. 2022). The full list of tracts is provided in Table 2

Building upon this approach, we previously devised tractography protocols for 42 WM tracts (FSL-XTRACT) that are standardised not only within species, but across very diverse brains, including humans and macaques (Mars et al. 2018b; Warrington et al. 2020), as well as adult and neonatal brains (Warrington et al. 2022) (Fig. 1). These protocols are defined in an analogous manner across brains and allow extraction of homologous WM bundles that exist across these different species, but are different in terms of their geometry, shape, exact location and projection patterns to grey matter. Subsequently, we have used these tracts as WM landmarks to anchor a common space and employed the uniqueness of cortical connection patterns to these tracts to probe areal specialisation. This has enabled us to quantitatively study divergences and similarities between brains with very different geometries (Mars et al. 2021), relying on connectivity patterns (instead of geometrically driven alignments), with higher relevance to brain function than allometric measurements (Eichert et al. 2020; Glasser et al. 2016; Mars et al. 2018a, b; Passingham et al. 2002; Van Essen et al. 2013; Xu et al. 2020).

Our original tractography protocols for the macaque brain were developed using the F99 macaque template, a choice motivated by the data availability and development around this template (Folloni et al. 2019; Mars et al. 2018b; Van Essen and Dierker 2007; Warrington et al. 2020). F99 is a hybrid surface-volume single-brain atlas containing a large set of associated reference data, including 15 areal partitioning schemes (with a probabilistic architectonic map), and connectivity data, as well as links to the CoCoMac and Markov connectivity databases (Markov et al. 2014; Paxinos et al. 2000; Stephan et al. 2001; Van Essen et al. 2012; Van Essen and Dierker 2007). However, a diverse set of alternative macaque brain templates have become available, either single-brain (such as D99; (Van Essen and Dierker 2007)), or population atlases (such as NMT (Seidlitz et al. 2018), Yerkes (Donahue et al. 2016), INIA19 (Rohlfing et al. 2012)), that are becoming more commonly used (e.g. for reporting chemical tracing experiments), providing their own sets of advantages and limitations. For instance, single-subject templates (F99, D99) tend to be of higher resolution but are more biased to the individual animal’s anatomy. On the contrary, multi-subject templates (INIA, Yerkes, NMT), are more representative of the group average, thus better for group comparisons, but can be limited in their relative neuroanatomical representation by the registration method (McLaren et al. 2009; Van Essen et al. 2001). Similarly, for in vivo templates (F99, NMT, INIA) the resolution and contrast are usually lower than for ex vivo templates (Yerkes, D99), with overall a single-subject ex vivo template having the highest contrast and resolution (D99) (Reveley et al. 2016; Seidlitz et al. 2018). Templates also differ in terms of the available associated data. For example, NMT has an extensive set of atlases both cortical and subcortical at various parcellation levels making it a useful template for atlas-based work (Seidlitz et al. 2018). Similarly, tracer studies often use the INIA (Howells et al. 2018) and NMT (Xu et al. 2022) templates (and less so the F99), thus making other templates more useful if data integration is in order. Therefore, depending on the data available and the questions at hand, a different template may be more or less appropriate, making the translation of our methods to multiple templates a valuable step.

In this paper, we present the generalisation of the original F99 macaque XTRACT tractography protocol across five macaque brain templates (F99, D99, NMT, Yerkes, and INIA) to allow for greater flexibility in the questions these protocols can be used for. We took the approach of first aligning the existing protocols to new templates to provide a starting point and subsequently adjusting them in each space to ensure maximum equivalence across templates. This allowed us to also revise imperfections in the original F99 tractography protocols. We demonstrate robustness and strong similarity of tract reconstruction within individual animals using protocols from the five different template spaces in a number of ways, including: (a) correlations of the reconstructed tracts using each alternate template against those using the standard F99 template and also (b) agreement of within-bundle dMRI microstructure metrics. We further demonstrate equivalence in grey-to-white matter whole-brain connectivity patterns when using tracts reconstructed from different templates in two different tasks: (a) identifying homologue cortical regions between the human and the rhesus macaque and (b) predicting scalar whole-brain maps from humans to macaques. Results confirm similarity of all predictions regardless of the macaque brain template used, providing direct evidence for the generalisability of tractography protocols across the five macaque templates considered.

## Methods

**Fig. 2:**
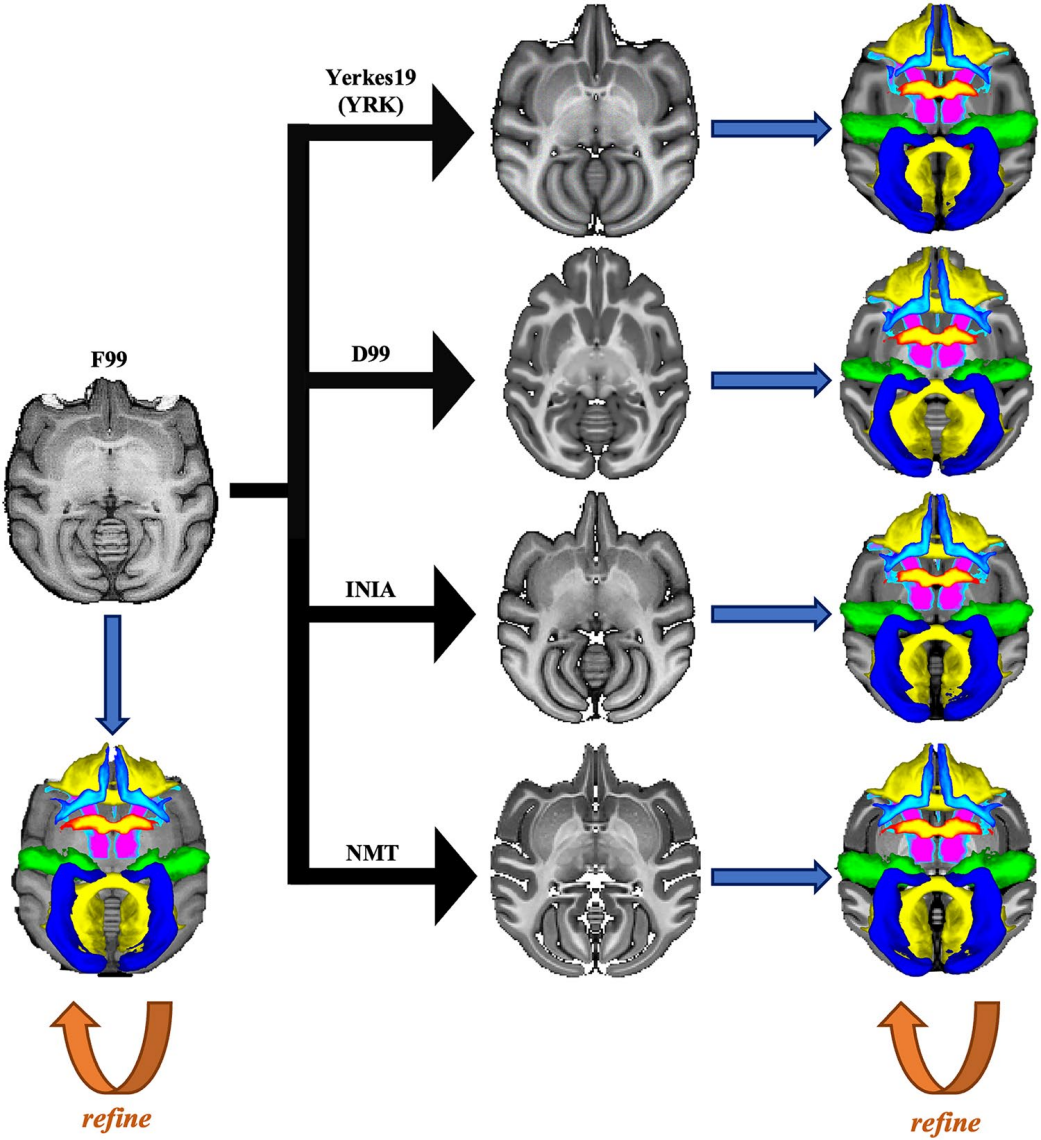
Nonlinear registration of our F99 protocol ROIs to each of the other template spaces. Iterative manual modification of ROIs within each template space was used, where needed, to obtain the final protocols. These protocols were used to produce population-averaged tract atlases in each template space

Our XTRACT tractography protocols comprise of regions of interest (ROIs) defined in F99 space that describe starting, exclusion, stopping, and waypoint criteria for 42 WM tracts. We transformed those to four new template spaces (Table 1), using the process summarised in Fig. 2. We first performed a non-linear registration between templates to obtain a starting point for transforming these protocols from F99 to the other four considered templates. Subsequently, we refined a number of them to ensure maximum generalisability. For example, a common challenge was induced left–right protocol asymmetry after registration in the new template spaces, which we had to manually adjust. Notably, we identified and adjusted F99 protocols that did not generalise well to other templates (for instance, comprising of small ROIs close to interfaces that were very sensitive to mis-alignment errors). The full list of tracts is shown in Table 2.

**Table 1:** All Macaque templates considered including the standard F99

Template Name	Species	Number of Animals	Reference	Resolution (mm)^a^	Field Strength	in vivo/ex vivo
F99	*M. mulatta*	1	Glasser and Van Essen (2011)	0.5 isotropic	4.7T	in vivo
INIA	*M. mulatta*	19	Rohlfing et al. (2012)	0.5 isotropic	3T	in vivo
D99	*M. mulatta*	1	Reveley et al. (2016)	0.5 isotropic	4.7T	ex vivo
NMT^b^	*M. mulatta*	31	Seidlitz et al. (2018)	0.25 isotropic	4.7T	in vivo
Yerkes19 (YRK)	*M. mulatta*	19	Donahue et al. (2016)	0.25 isotropic	4.7T	ex vivo

^a^All templates were resampled to isotropic 0.5 mm resolution (where necessary)

^b^We consider NMTv2.0 in this work, for simplicity we call it NMT

**Table 2:** The 42 WM tracts included in XTRACT, their type, full and abbreviated name

Category	Tract name	Abbreviation
Association fibres	Arcuate fasciculus	AF
	Frontal aslant tract	FA
	Inferior fronto-occipital fasciculus	IFO
	Inferior longitudinal fasciculus	ILF
	Middle longitudinal fasciculus	MdLF
	Superior longitudinal fasciculus I, II and III	SLF 1,2,3
	Uncinate fasciculus	UF
	Vertical occipital fasciculus	VOF
Commissural fibres	Anterior commissure	AC
	Forceps major (splenium of the corpus callosum)	FMA
	Forceps minor (genu of the corpus callosum)	FMI
	Middle cerebellar peduncle	MCP
Limbic fibres	Cingulum bundle (CB): dorsal section	CBD
	CB: perigenual section	CBP
	CB: temporal section	CBT
	Fornix	FX
Projection fibres	Acoustic radiation	AR
	Anterior thalamic radiation	ATR
	Corticospinal tract	CST
	Optic radiation	OR
	Superior thalamic radiation	STR

Apart from the commissural tracts, all other tracts are bilateral and have separate protocols for their left and right counterparts

Specifically, we used the RheMAP approach (Klink and Sirmpilatze 2020) to calculate non-linear warp fields between the F99 space and the other four template spaces. These templates were based on both single-subject and group-averaged, in-vivo and ex-vivo anatomical (T1w) MRI data and included: D99 (Reveley et al. 2016), INIA (Rohlfing et al. 2012), NMTv2.0 (which will be referred to as NMT) (Seidlitz et al. 2018), Yerkes19 (YRK) (Donahue et al. 2016). RheMAP uses ANTs (Avants et al. 2010, 2011) to generate non-linear warp fields, which we converted to FSL format. These non-linear warp fields were then used to transform the F99-space protocol ROIs (for the 42 tracts) to each of the new template spaces (Table 2). This provided a good starting point for the protocols in the new template spaces. Where registration caused imperfections (such as in the case of the perigenual section of the cingulum bundle (CBP), the corticospinal tract (CST), and the anterior commissure (AC)) manual revisions of the protocols were performed in all five template spaces. The revisions involved modification of exclusion, seed and target ROIs, to achieve bilateral symmetry, as well as to avoid ROI overlap and eliminate mapping outside the brain. Thus, we achieved equivalent protocols in all template spaces. Twelve of the original 42 protocols required substantial adjustment.

### Data

*Macaque*: we used ex vivo dMRI brain data of 6 rhesus macaques (4–16 years of age), publicly available from PRIME-DE (http://fcon_1000.projects.nitrc.org/indi/PRIME/oxford2.html) (Milham et al. 2018). Data were acquired using a 7T Agilent DirectDrive console, with a 2D diffusion-weighted spin-echo multi-shell (DW-SEMS) protocol (16 volumes acquired at *b* = 0 s/mm^2^, 128 volumes acquired at *b* = 4000 s/mm^2^), at 0.6 mm isotropic resolution.

*Human:* Minimally preprocessed dMRI data (Glasser et al. 2013; Sotiropoulos et al. 2013) from the young adult Human Connectome Project (HCP) (Van Essen et al. 2013) were used for the assessment of cross-species comparisons across the different macaque template spaces. Data from 50 unrelated subjects (age range, 22–35 years), (3T 1.25 mm isotropic resolution data, with 270 directions evenly distributed across *b* = 1000, 2000 and 3000 s/mm^2^ shells) were used.

### Preprocessing, tractography and population white matter (WM) tract atlases

Animal-wise non-linear transformations to the F99 template space were obtained using FSL’s FNIRT (Andersson et al. 2007), using each animal’s FA maps. Animal-wise non-linear transformation fields to each of the other four template spaces were obtained by concatenating the animal-wise F99 warp fields and the warp fields between the F99 and every other template space (FSL format of RheMAP non-linear transforms described above) using the FSL package tools (Jenkinson et al. 2012).

Prior to tractography, for each animal, fibre orientations were estimated (up to three per voxel) using FSL’s BEDPOSTX (Hernández et al. 2013; Jbabdi et al. 2012). Using the template-specific protocols, probabilistic tractography was performed using FSL’s XTRACT (Warrington et al 2020). A curvature threshold of 80° was chosen, and subsidiary fibres were considered with a volume fraction greater than 1%. A maximum of 2000 streamlines steps were used, and a step size of 0.2 mm was chosen. The resultant distributions of streamline counts were normalised by the total number of valid streamlines generated (those not rejected by inclusion/exclusion mask criteria) and stored in each subject’s respective native space, representing the corresponding path distributions.

**Fig. 3:**
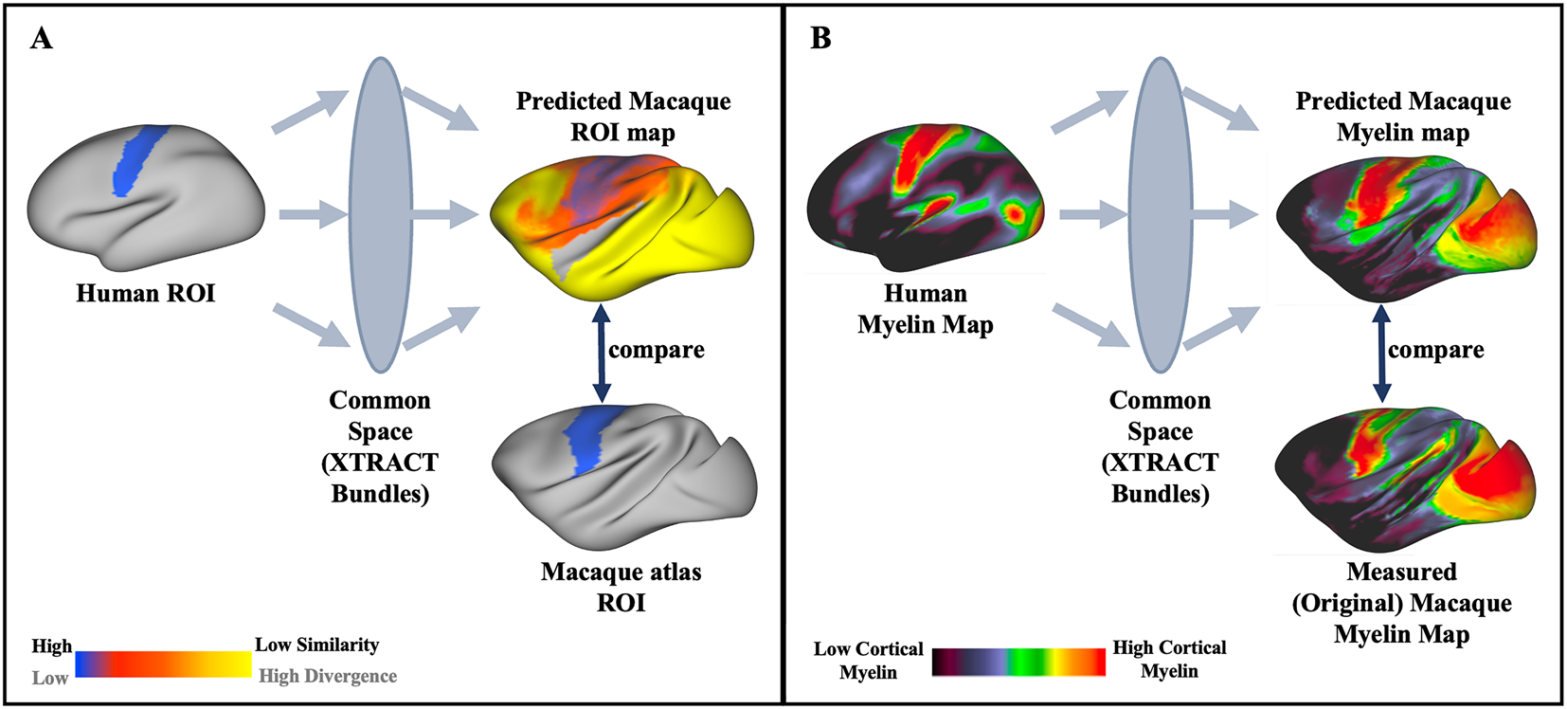
KL divergence in our WM-anchored common space gives the (dis-)similarity between the connectivity patterns of cortical parcels across brains and allows for identification of corresponding cortical regions between the human and the macaque. **A** Minimisation of KL divergence maps to find the corresponding region in the macaque brain to human cortical ROIs (sensorimotor area shown here). **B** Use KL divergence maps to project the human myelin map to the macaque brain

Tractography results of all animals per template were used to obtain a set of tract atlases per template, in the form of population percentage overlap. For each template space, the respective native-space normalised path distributions were transformed to the template space, binarised and averaged across animals. The resultant spatial maps describe the percentage of animals for which a given tract is present at a given voxel. These maps were used for qualitative comparison of the tractography output between F99 and each new template (Fig. 3).

### Protocol assessment

The robustness of the protocols and the generalisability of our framework to the other template spaces were assessed.

#### Comparing tract similarity across templates

For each animal, we calculated, per WM tract, the correlation between the native-space path distribution obtained using the F99 protocol and the native-space path distribution obtained using the protocol for every other template (INIA, NMT, Yerkes, D99). To do so, each 3D path distribution was turned into a 1D vector and pairwise Pearson’s correlation between the corresponding vectors were computed. Then, we summarised the correlation between each template and F99 per WM tract as the mean ± std (standard deviation) across animals. Within-tract measures of microstructure — tract-wise fractional anisotropy (FA), tract-wise mean diffusivity (MD) — were also compared across templates, exploring whether the new protocols localise equivalent WM areas. To obtain such measures, path distributions for each tract were normalised and thresholded at 0.1% giving rise to a binary mask per tract and template. The median value of FA and MD within each of these masks was obtained.

#### Comparing similarity of connectivity patterns across templates

Having extracted WM bundles across template spaces, we explored their equivalence in performing whole-brain comparisons between the human and macaque brains. Following the common space approach (Mars et al. 2018b), we extracted five versions of whole-brain grey-to-white matter connectivity patterns, one version corresponding to each of the considered macaque template spaces. For each of them, human and macaque comparisons were performed for two applications (scalar map prediction and homologue area identification, Fig. 3) and similarity of results across templates was evaluated. These comparisons allowed us to explore any differences between the proposed protocols in a complementary way to the previous pairwise similarities, which can capture the main body of a bundle, but less so any subtle deviations of the projections of these bundles.

To perform the above comparisons, we first built connectivity blueprints of the human and macaque brain (Mars et al. 2018b). These are whole brain (grey matter × WM tracts) matrices and we used the aforementioned 42 XTRACT tracts (Warrington et al. 2020). Specifically, tractography results for all 42 tracts were unwrapped in 1D, yielding a (whole brain × tracts) matrix. Whole-brain probabilistic tractography was performed to build a (cortex × whole brain) connectivity matrix, seeding 1000 streamlines from the white matter–grey matter boundary (WGB) and counting visitations in a whole-brain mask with the ventricles removed, down-sampled to 2 mm^3^. Connectivity blueprints (cortex × tracts) were then obtained by taking the product of this whole-brain connectivity matrix and the vectorised tract matrix. The columns of the resulting matrix represent the projection patterns of each tract on the WGB surface, whereas the rows represent the connectivity pattern of each of the WGB locations.

Surface data in F99 space were obtained following the approach described in Mars et al. (2018b). Briefly, a single set of macaque surfaces were derived using a set of high-quality structural data from one of the macaque subjects. The remaining macaque data were then non-linearly transformed to this space, and the surfaces were non-linearly transformed to the F99 standard space (Van Essen 2002). Prior to tractography, the surfaces were downsampled to approximately 10,000 vertices per hemisphere. Surfaces were only obtained in the F99 template space. For the other four template spaces, the template-specific tracts were non-linearly transformed in the F99 space and the above approach was followed to obtain connectivity blueprints using the F99-space surfaces. Following subject-wise construction of connectivity blueprints in each template space, we derived group-averaged blueprints for each template space, as well as one group-average blueprint for the human brain.

Once connectivity blueprints for the human and macaques are obtained, normalised rows of these matrices can be compared across species, as they correspond to patterns of connections of different grey matter locations to analogously defined WM tracts. We used this approach to identify the macaque homologues of two exemplar human brain areas (Fig. 3A) and explored the similarity when using tracts and blueprints obtained across the different macaque templates. We used the Glasser cortical atlas (Glasser et al. 2016) to define the human primary sensorimotor area (M1 + S1) and the medial temporal area (MT). For each of these areas, the average connectivity profile was extracted, by averaging the rows of the blueprint corresponding to locations within each of the areas, respectively. Subsequently, we looked for the vertices on the macaque cortical surface with the greatest similarity to the human connectivity profile for the same tracts. We used the symmetric Kullback–Leibler (KL) divergence (Kullback and Leibler 1951; Mars et al. 2018b) to assess similarities. KL divergence is zero for equivalent profiles and increases as they diverge. Hence, looking for the set of locations in the macaque with the minimum KL-divergence against the selected human brain areas allowed us to identify the macaque homologues of M1 + S1 and the MT. We compared the identified locations with reference regions obtained from the Paxinos macaque atlas (Paxinos et al. 2000) and we repeated this process independently for the five macaque template spaces. In all cases, we excluded the insula (Glasser atlas for the human and a combination of the M132 and MMF11 Markov atlases for the macaque brain (Glasser et al. 2016; Markov et al. 2014, 2011)), as it was poorly represented by the set of tracts reconstructed.

As a second step, we used the human-macaque similarities and divergences in the patterns of connectivity to our predefined set of tracts to project a scalar map (myelin maps (Glasser and Van Essen 2011)) from one species to the other (Fig. 3B). Effectively, we used connectivity patterns as a warp field and explored how tracts from the different macaque templates affect this transformation. To do so, for each of the macaque template spaces, we obtained a dis-similarity matrix to the human using the corresponding macaque and human blueprints. For each WGB location on the macaque brain we found the KL divergence across all WGB locations in the human. This KL divergence matrix was used to project the human myelin map (group average obtained from the HCP) to the macaque brain for each of the macaque templates (Fig. 3B), as described in (Mars et al. 2018b). Consistency of the myelin predictions across templates and with respect to the original F99 template, reinforces the generalisability of our framework to the other template spaces.

## Results

**Fig. 4:**
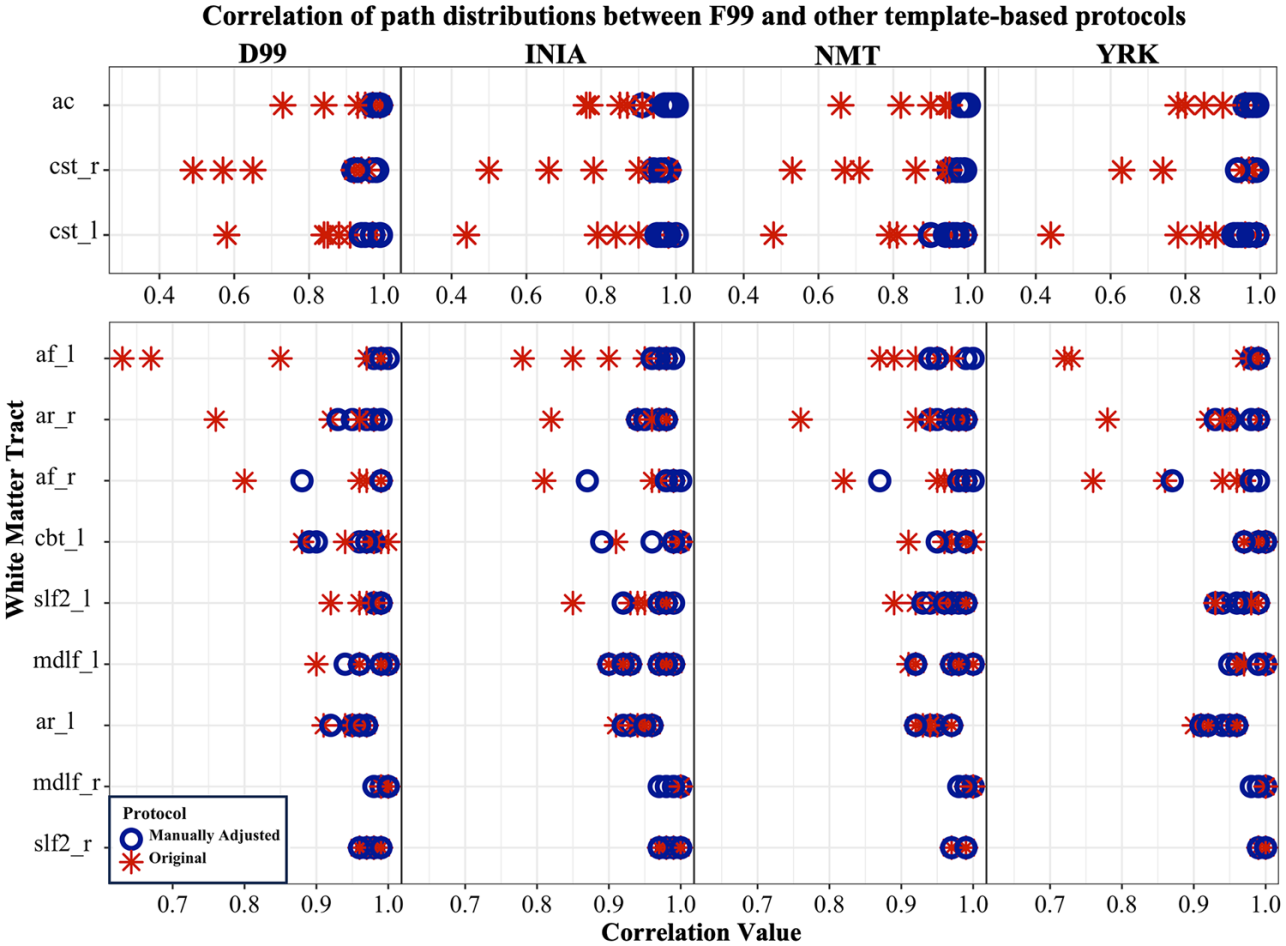
Subject-wise correlation values between path distributions of tracts reconstructed using the F99-based protocols and each of the other template-based protocols. A subset of 12 (of the 42) tracts is shown comprising of the ones that required manual refinement following registration from the F99 template. For each tract and template, six samples are shown for “Original” (red asterisks, protocols are simply the F99 ones registered to new template space) and six for “Manually Adjusted” (blue circles, protocols have been refined in each template space), corresponding to the six macaque datasets we used. Adjustment was performed to improve tractography results and increase consistency across templates

We first assessed the robustness of tractography protocols that were directly registered from F99 to other template spaces. By assessing correlations of the resultant tracts between template spaces, we found that for 12 tracts the original F99 protocols did not generalise well when simply registered through non-linear warps (Fig. 4). We used large differences in correlation and qualitative inspection of the tracts as evidence that protocols needed further revision. We performed such revisions in two ways, both for the protocols in the new templates, but also for the original F99 protocols, when needed. Specifically, the original F99 protocols were modified for 6 tracts: CST (left), AR (right), AC, MDLF (right and left), CBT (right). In all tracts the seed, target and exclusion ROIs were modified, as detailed in Table 3.

**Table 3:** Changes in the ROIS of the original F99-based protocols

	Seed	Target	Exclude	Stop
CST_left_	Expanded the pontine seed medially and removed a few lateral voxels close to the boundary to avoid being outside the brain after registration	Expanded the cortical target to cover greater cortical area, but also removed a few superior voxels close to the boundary to avoid being outside the brain after registration	Shifted to better exclude midline; removed voxels along boundary to the seed to avoid overlap (before and after registration)	*(No change)*
AR_right_	Mirror transverse temporal gyrus seed from the left and added to the original right, and slightly expanded avoiding gyri and exclusion	*(No change)*	Mirrored exclusion from the left, binarised and modified to avoid seed overlap	*(No change)*
AC	Largely expanded and lateralised left–right midline fibres seed to maintain good size after registration (tracking in the left was better than in the right side)	Largely expanded and lateralised left to right to maintain good size after registration (tracking in the left was better than in the right side)	Eroded by one voxel along border with seed to avoid overlap. Expanded dorsally to eliminate false positives	Lateralised left to right (tracking in the left was better than in the right side)
MDLF_left_	*(No change)*	*(No change)*	Improved NMT exclusion registered to F99 and modified to avoid overlap to seed and target	*(No change)*
MDLF_right_	Expanded superior frontal gyrus seed to increase reproducibility across subjects	*(No change)*	*(No change)*	*(No change)*
CBT_right_	*(No change)*	*(No change)*	*(No change)*	Mirrored left side

**Fig. 5:**
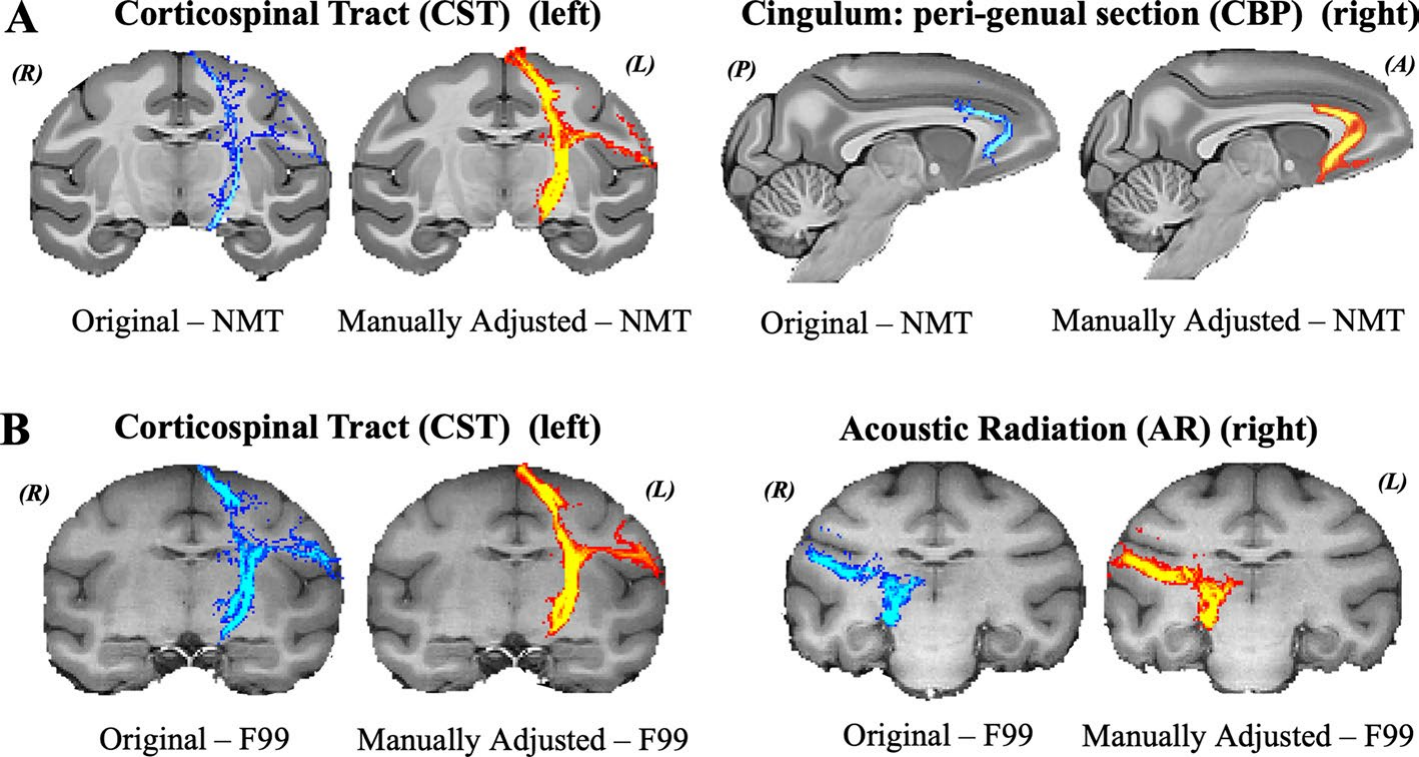
Examples of tracts obtained using protocols that were manually adjusted following the nonlinear registration from F99 to each other template space. **A** CBP and CST in NMT space, before (denoted as “Original”) and after adjustment of the registered protocol ROIs (from F99 space) (denoted as “Manually Adjusted”). **B** AR and CST in F99 space, before and following adjustment. Coronal sections are shown using radiological convention: *R* right, *L* left, *A* anterior, *P* posterior

This iterative process resulted in high agreement between within-animal tractography results when using the five different templates. Figure 5 demonstrates representative examples of tracts obtained from the same animal, when using the original F99 protocol registered to a new template (“Original”) vs the refined protocols (“Manually Adjusted”). Figures 4 and 7 highlight the improvement such revisions made across the six different animals and across the considered templates.

### Tract similarity across template spaces

**Fig. 6:**
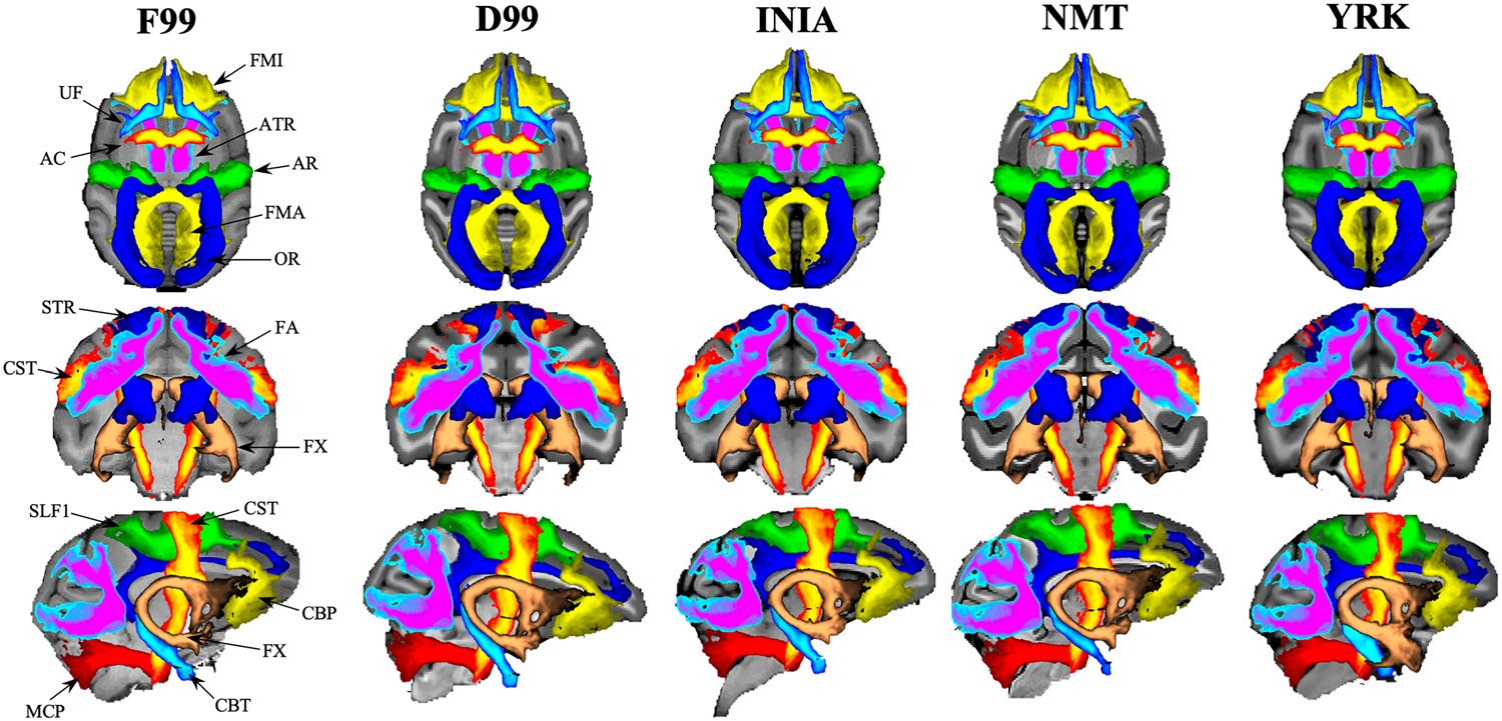
Group-average WM tracts from the final protocols using the five templates (F99, D99 INIA, YRK, NMT). Qualitative comparison between templates for all major tracts considered. For each tract, normalised path distributions were thresholded at 0.1% and binarised, and then averaged across the six animals. The above maps show group percentage averages of these distributions (colour codes depict from 30% to 100% of the group)

**Fig. 7:**
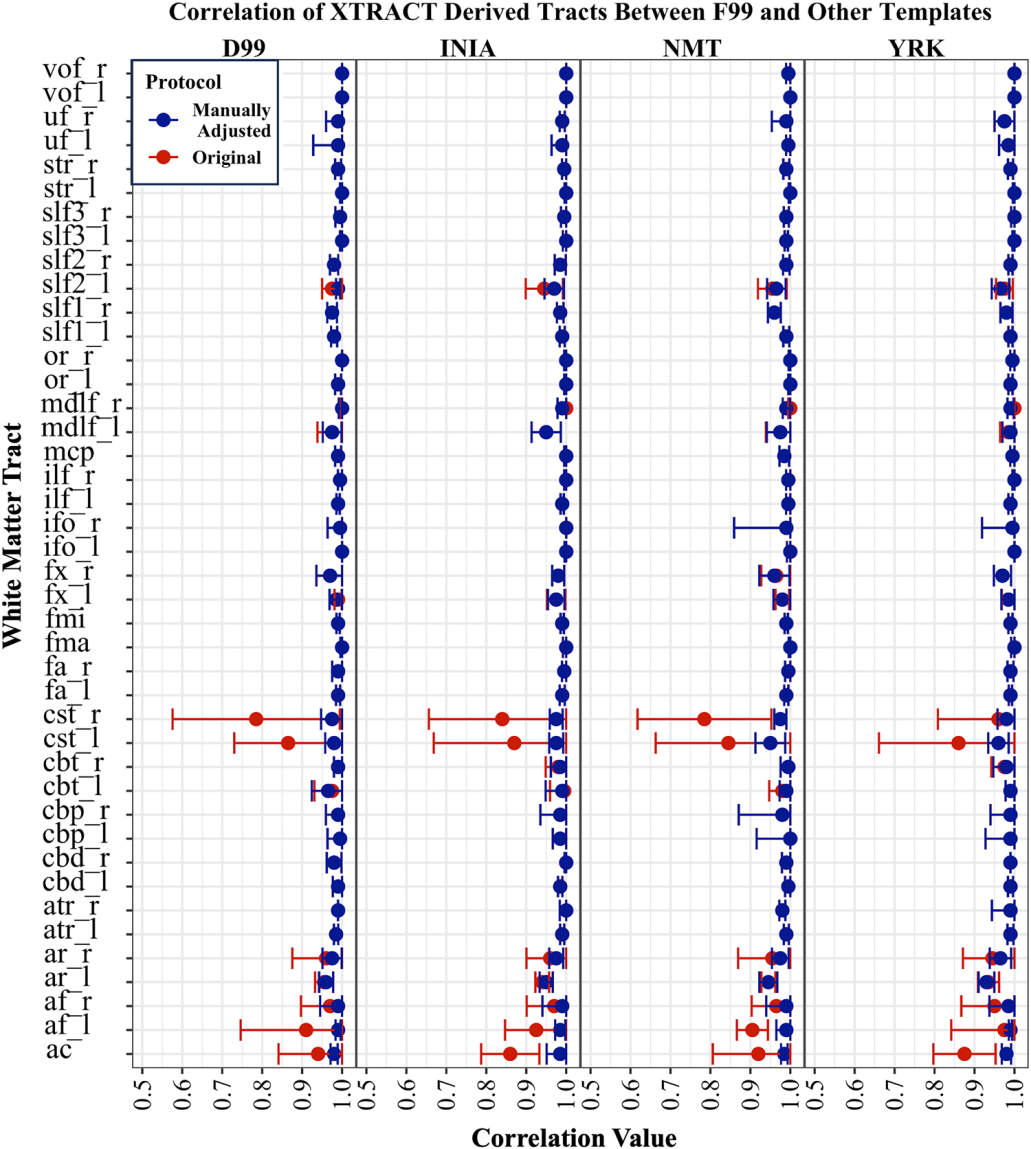
Correlation (mean ± std) across six subjects for each tract between F99-derived path distributions and those derived using each other template considered. The correlation values were used for template comparison and assessment of protocol robustness and generalisability. For each tract and template space, normalised path distributions were thresholded at 0.1% and the Pearson correlation against the F99 respective path distribution was obtained within a binary mask covering the F99 thresholded path distribution

**Fig. 8:**
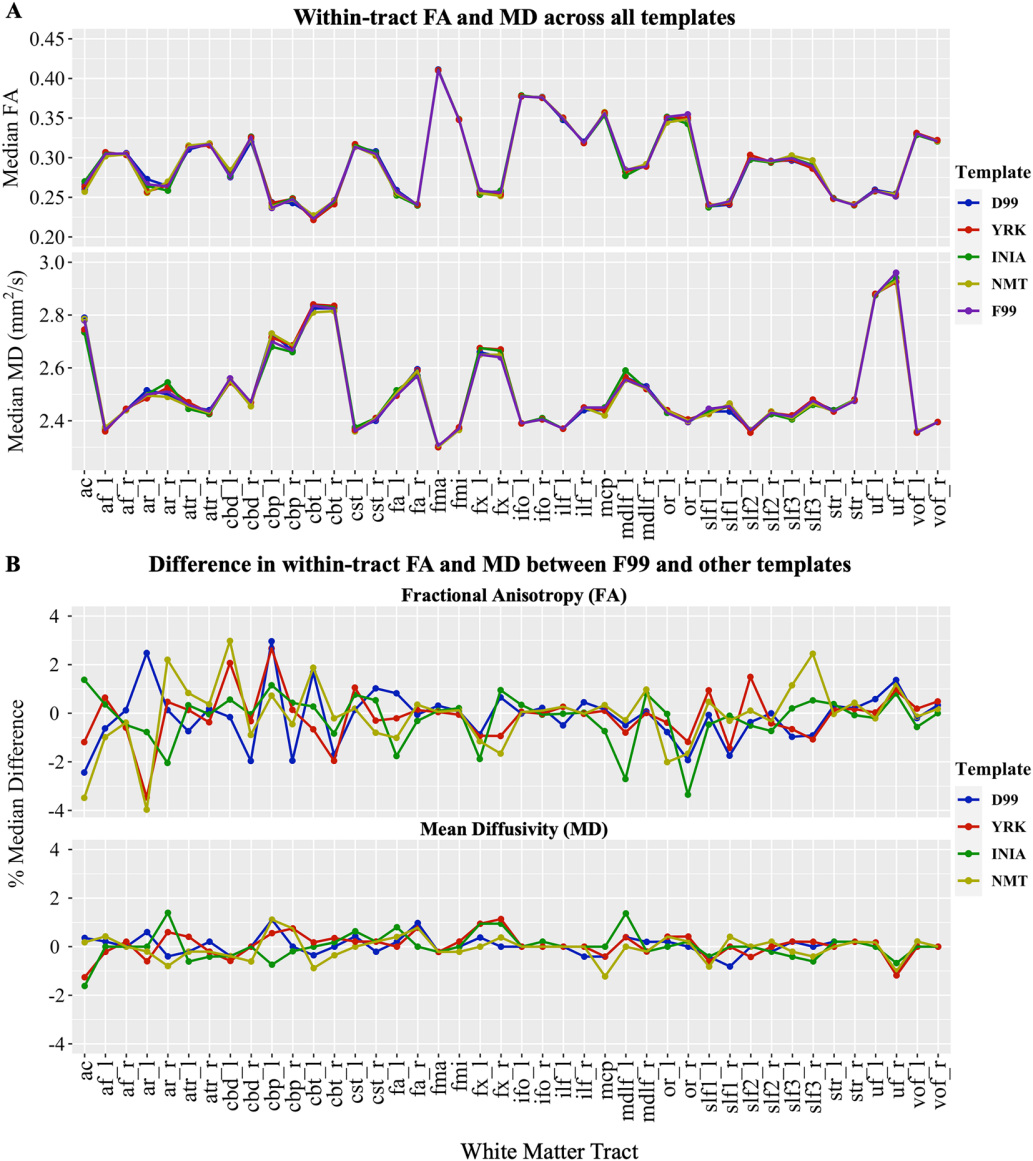
Within-tract diffusion measures of microstructure — tract-wise fractional anisotropy (FA), tract-wise mean diffusivity (MD) — were used to assess protocol robustness and generalisability across templates, as more biologically relevant measures compared to correlation. **A** The median FA and MD values per WM tract across subjects for each template space. Values are comparable across templates. **B** Percent difference of FA and MD of each new template considered compared to F99. The majority of absolute differences are less than 2%

Qualitatively, we observed high agreement between the path distributions generated using the standard F99 and the corresponding protocols in other template spaces across animals (Fig. 6). To quantify the agreement, we calculated the mean correlation ± std per tract across animals, between F99 and every other template. For all 42 WM tracts, median correlation was greater than 0.9 between F99 and every other template across animals. For each tract category (association, commissural, limbic, projection), the median agreement between the F99 and the in-vivo templates (INIA19, NMT) was ~ 0.99, as well as for the ex-vivo templates (D99, YRK) (Fig. 7). Complementary to the tract correlations, we also assessed within-tract DTI values (FA, MD). Both were consistent across all five templates considered, with the differences being mostly within 2% of the values obtained using the F99-based tractography protocols (Fig. 8).

### Similarity of connectivity patterns across templates

**Fig. 9:**
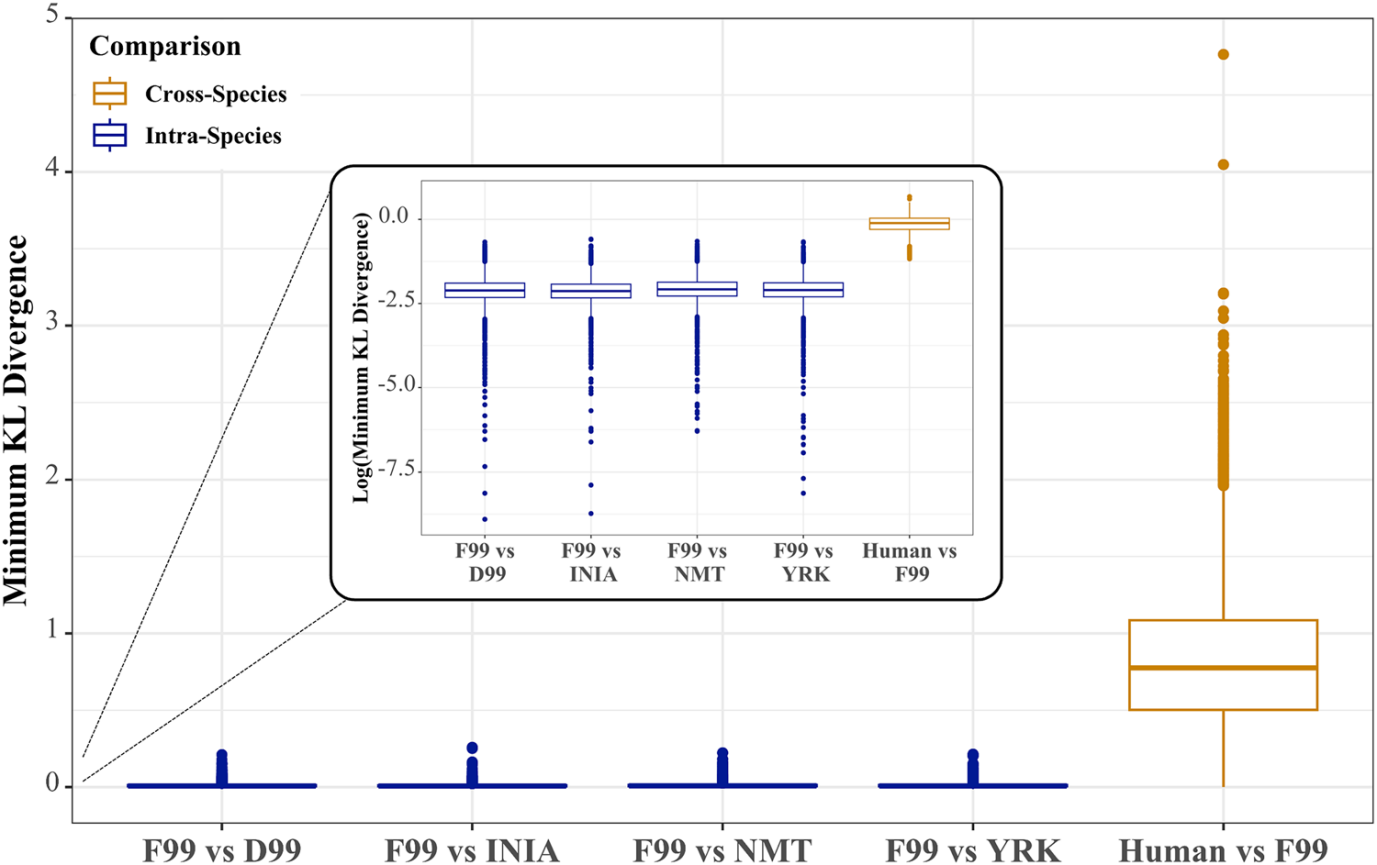
Distributions of minimum KL divergence quantifying the connectivity-based (dis-)similarity between F99 and each other macaque template blueprint, as well as with the human blueprint. For each WGB location in F99 space, the divergence with the best matching vertex in other template spaces (i.e. minimum KL divergence) has been calculated. The boxplots represent the distribution of these minimum KL divergence values across the whole brain. The inset is the log_10_ of the same KLD values. The cross-species dis-similarity (i.e. between the human and the macaque) strongly surpasses the intra-species dis-similarity (i.e. amongst macaque templates)

In addition to ensuring similarity in terms of the bodies of the tracts across templates, we also explored similarity in the projection patterns of these tracts. KL divergence was calculated between average human and macaque connectivity blueprints (for every template space) to obtain (dis-similarity matrices between the macaque and human connectivity patterns. We also calculated the KL divergence between macaque connectivity blueprints obtained using the F99 protocols and the protocols from the other template spaces. Figure 9 demonstrates that these divergences are very small (almost zero, median around 0.008 in all cases) compared, for instance, to macaque-human divergences which are on average two orders of magnitude higher (median 0.8). This provides evidence that projections patterns of individual tracts are very similar regardless of the protocols used.

**Fig. 10:**
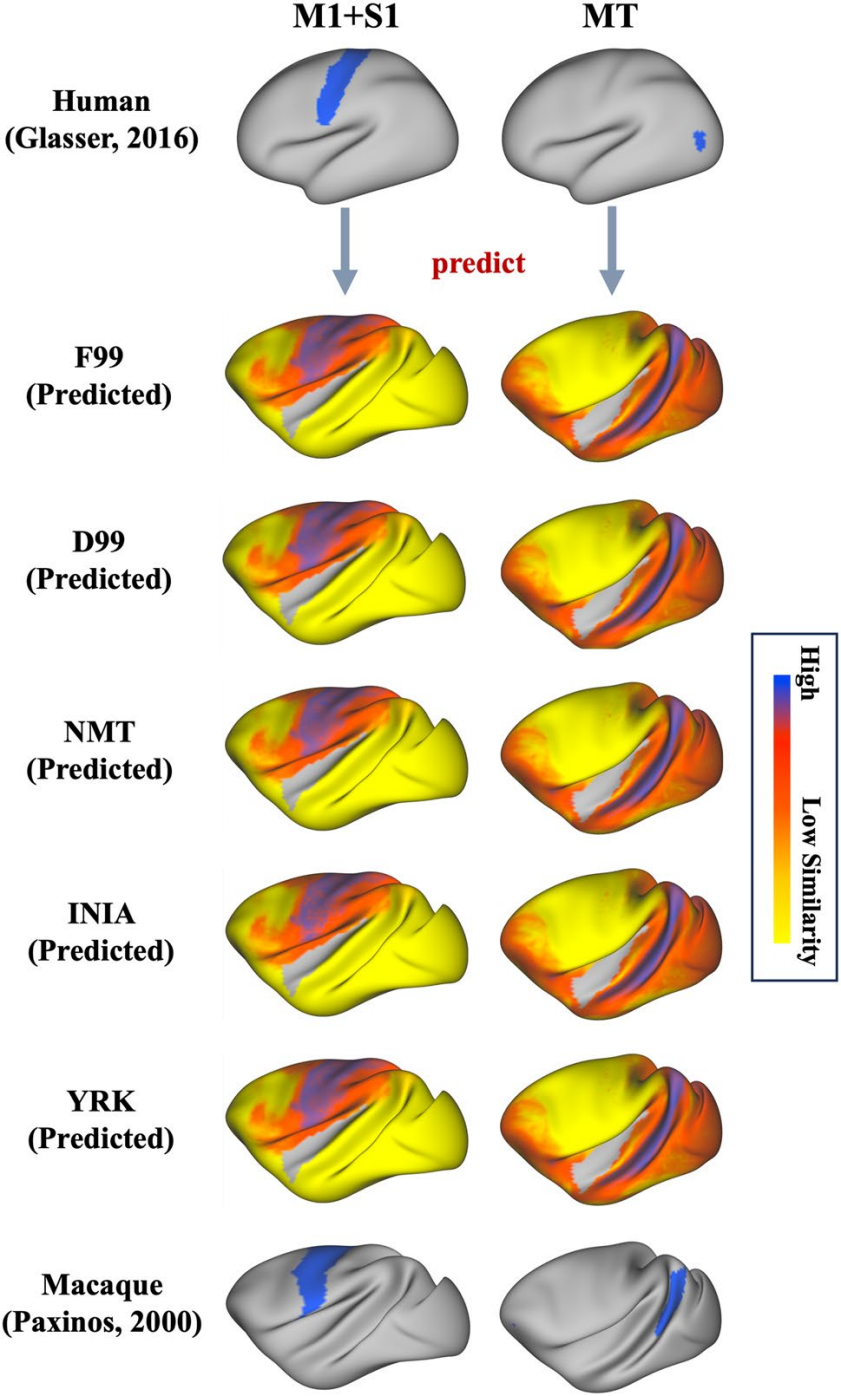
KL divergence maps quantifying the connectivity-based (dis-)similarity between two human regions (primary sensorimotor-left and MT-right) and the macaque brain, when using tracts defined by protocols across the different macaque templates. Macaque regions with the most similar connectivity patterns to the human regions (i.e. lowest KL divergence) are shown in purple. The macaque primary sensorimotor and MT areas from the Paxinos atlas are shown for reference (bottom row). In all template spaces, the resulting patterns closely resemble the expected location of the corresponding regions in the macaque, with regions of lowest divergence overlapping with the atlas-based ROIs for M1 + S1 and MT respectively

We further explored whether any small differences in the projection patterns have an effect on downstream analyses. We performed two human-macaque comparisons on the basis of their connectivity patterns to the correspondingly defined WM tracts. Firstly, we identified the most similar cortical vertices from the macaque brain to the average connectivity pattern of two exemplar regions of the human brain, the primary sensorimotor area (M1 + S1) and the medial temporal area (MT), recapitulating previous work (Mars et al. 2018a, b; Warrington et al. 2022). Figure 10 demonstrates the corresponding KL-divergence values for both regions when using tracts reconstructed from protocols of the different macaque templates. We found high agreement in the cross-species cortical area mapping for the F99, and the other macaque template spaces considered, supporting the generalisability of the protocols to the new template spaces.

**Fig. 11:**
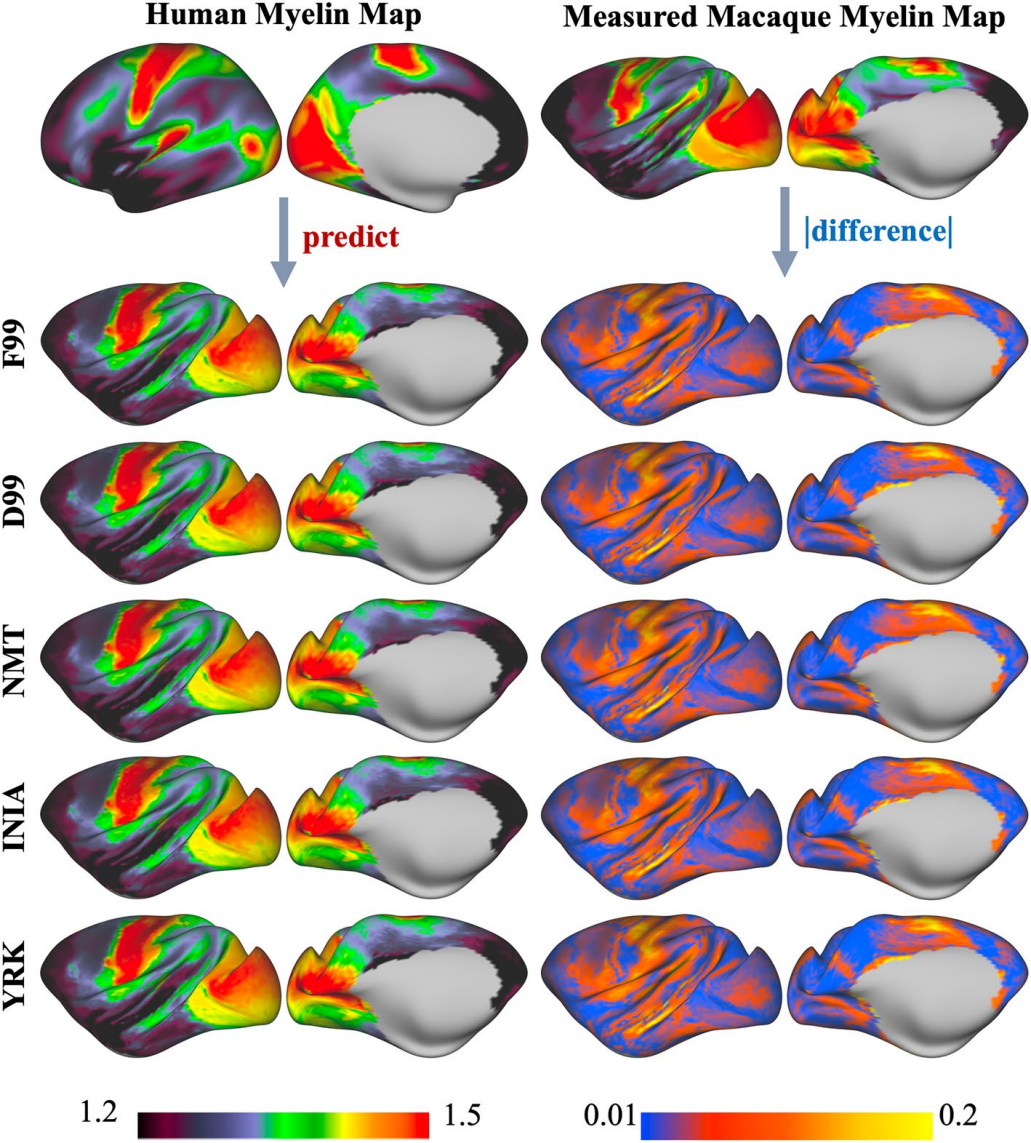
Prediction of macaque myelin map from a measured (T1w/T2w ratio) human myelin map for each template space considered (left). The KL-divergence of connectivity patterns to the reconstructed WM tracts between human and macaque is used as a transformation field. The right column shows absolute difference between each myelin map prediction and a measured (T1w/T2w ratio) macaque myelin map

Secondly, whole-brain KL divergence matrices (i.e. matrices that depict for every vertex in the macaque brain the best agreement with the human ones) were used to project the human myelin map to the macaque brain for each of the macaque templates. Figure 11 demonstrates that the myelin prediction maps for each macaque brain were equivalent, each having a very similar correlation of ~ 0.84 to the measured (original) macaque myelin map. The pattern of the difference maps between predicted and measured is also equivalent against template spaces. With this step we do not only ensure generalisability of the tractography protocols across templates (agreement in the results across templates) but also the reliability of these results due to their high correlation to the measured myelin map.

Taken together, these results support the robustness of the protocols to macaque template. As these new macaque tractography protocols are also correspondingly defined for the human brain, this expansion allows for greater flexibility and integration of the WM-based common space framework for probing brain differences and similarities across evolution and development (Mars et al. 2018b; Warrington et al. 2022).

## Discussion

In this work, we presented generalised XTRACT tractography protocols across five different commonly used macaque brain template spaces, specifically F99, D99, INIA19, Yerkes, NMT. We aimed to define protocols for 42 WM tracts that provided equivalent tract reconstructions within the same animal, regardless of the template space used. We demonstrated such equivalence through a number of tests, ranging from similarity metrics that focussed on the body of the tracts to similarity tests of the projection patterns of each tract. As these tractography protocols are also correspondingly defined for the human brain (Warrington et al. 2020), this generalisability allows for greater flexibility and integration of our previously presented WM-based common space framework for probing brain differences and similarities across phylogeny (Mars et al. 2018b; Warrington et al. 2022). This also allows for easier integration of our WM connectivity data with other data types, thus harvesting the power of multimodal data in addressing more complex neuroanatomical questions. For example, in combination with histology or tracer data, which can be available in any of the other common template space, we can better target the biological underpinnings of the structural connectivity features we observe. Similarly, there can be great benefit from integrating structural with functional connectivity information to better elucidate the crosstalk between different brain regions, both within and across species (Garin et al. 2022; Zimmermann et al. 2018).

We used an iterative process involving the manual refinement of the original F99 protocol ROIs, the non-linear registration to every other template space and the subsequent manual refinement in each template space of protocols where the registration caused issues (such as ROI overlap, ROI expansion beyond the brain mask, ROI left–right asymmetry). This process resulted in reproducible tracking for all 42 tracts considered in all template spaces, with no further manual adjustment required by the user. Specifically, within-animal path distributions, produced using protocols based on macaque templates other than F99, were highly correlated (mean correlation ≥ 0.9) to those obtained using the standard F99 template. Furthermore, we looked at within-tract mean DTI measures (FA and MD) and found very high consistency across all five templates, indicating that all protocols resulted to almost identical tract localisation.

Connectivity blueprints were subsequently used to explore whether using protocols from other than F99 templates causes changes in the connectivity patterns. We used connectivity patterns to reconstruct WM tracts to perform two human-macaque comparative experiments: one identifying homologue areas between human and macaque brains using similarity in connectivity patterns for localising functionally similar regions across species; and one using connectivity patterns to obtain a transformation for mapping cortical maps across two species. Equivalence in reconstructed tracts across macaque template spaces would result into equivalence of results in these two experiments. Indeed, results were consistent across all five templates and in agreement with previously published results (Mars et al. 2018b; Warrington et al. 2022). These complementary analyses indicated that the more subtle projection patterns of the reconstructed tracts are also equivalent across templates.

Although we have extended XTRACT protocols to five commonly used macaque template spaces, we recognise that there are still more templates available, so it would be of great interest and utility to further generalise our framework to even more template spaces (Van Essen 2002). It would also be beneficial to similarly extend to other NHP species and templates, with a natural extension being on the protocols for which we already have a first version, including the chimpanzee (Bryant et al. 2020), the gibbon (Bryant et al. 2023), the gorilla (Roumazeilles et al. 2020) and the marmoset (Schaeffer et al. 2017). This will allow for even greater flexibility in the questions our framework can be used to address. This work would not have been possible without the dedication of many in the NHP community to data collection, standardisation, documentation, and sharing (Milham et al. 2020, 2022). It is only through open science and sharing of our data and expertise that we can overcome shared problems and push the boundaries of the field. We aim with our work, including the one presented here, to contribute to this goal moving forward.

## Data Availability

Macaque data used in this study are publicly available from PRIME-DE (http://fcon_1000.projects.nitrc.org/indi/PRIME/oxford2.html) (Milham et al. 2018). Human data are publicly available from the young adult Human Connectome Project (HCP) (https://www.humanconnectome.org/study/hcp-young-adult). Tractography protocols and template-specific tract averages for all five template spaces are available on (https://github.com/SPMIC-UoN/XTRACT_Macaque_MultiTemplate) and will be incorporated into FSL-XTRACT (https://fsl.fmrib.ox.ac.uk/fsl/fslwiki/XTRACT).
